# Intersections Between Systems Thinking and Market Shaping for Assistive Technology: The SMART (Systems-Market for Assistive and Related Technologies) Thinking Matrix

**DOI:** 10.3390/ijerph15122627

**Published:** 2018-11-23

**Authors:** Malcolm MacLachlan, Joanne McVeigh, Michael Cooke, Delia Ferri, Catherine Holloway, Victoria Austin, Dena Javadi

**Affiliations:** 1 Department of Psychology, John Hume Building, North Campus, Maynooth University, Maynooth, Co. Kildare, Ireland; Joanne.McVeigh@mu.ie (J.M.); michael.cooke@mu.ie (M.C.); 2 Assisting Living & Learning (ALL) Institute, Maynooth University, Maynooth, Co. Kildare, Ireland; delia.ferri@mu.ie; 3 Centre for Rehabilitation Studies, Faculty of Medicine and Health Sciences, Stellenbosch University, P.O. Box 241, Cape Town 8000, South Africa; 4 Olomouc University Social Health Institute (OUSHI), Palacký University, Univerzitní 22, Olomouc 771 11, Czech Republic; 5 Department of Law, New House, South Campus, Maynooth University, Maynooth, Co. Kildare, Ireland; 6 Centre for European and Eurasian Studies, Maynooth University, Maynooth, Co. Kildare, Ireland; 7 UCL Interaction Centre (UCLIC), University College London, 66-72 Gower Street, London WC1E 6EA, UK; c.holloway@ucl.ac.uk; 8 Global Disability Innovation Hub, UCL at Here East, 8-9 East Bay Lane, Queen Elizabeth Olympic Park, London E15 2GW, UK; victoria.austin@ucl.ac.uk; 9 The Alliance for Health Policy and Systems Research, World Health Organization, 20 Avenue Appia, 1211 Geneva, Switzerland; javadid@who.int

**Keywords:** systems thinking, market shaping, assistive technology, assistive products, resource poor settings, low- and middle-income countries

## Abstract

The Sustainable Development Goals (SDGs) aspire to “leave no-one behind”. Universal access to assistive products is a critical link between the realization of the SDGs and those most likely to be left behind. However, assistive technology provision in many countries, particularly low- and middle-income countries, has traditionally been conducted through small-scale local providers, manufacturing products of varying degrees of quality at a limited price range. An effective way to scale these production and provision enterprises to the required level is needed to close the gap between available and required assistive technology. We argue that better access to assistive technology will only be realized through the adoption of a far stronger systems thinking and market shaping approach. We undertook a rapid literature review to explore the relationship between market shaping and assistive technology. Based on our review, we present an emergent framework for conceptualizing intersections between systems thinking and market shaping for assistive technology—the SMART (Systems-Market for Assistive and Related Technologies) Thinking Matrix.

## 1. Introduction

### 1.1. Background

The 2030 Agenda for Sustainable Development affirms that “no-one will be left behind” (p. 1), aspiring to “a just, equitable, tolerant, open and socially inclusive world in which the needs of the most vulnerable are met” (p. 4) [1]. In light of this, the World Health Assembly declares that including assistive technology (AT) in health systems is critical to achieving progress towards the targets of the Sustainable Development Goals (SDGs) including universal health coverage, inclusive quality education, inclusive and sustainable economic growth, and decent work for all, especially for persons with disabilities [2]. Assistive products (APs) are vital for the realization of each of the SDGs, and universal access to APs is a critical link between the realization of the SDGs and those most likely to be left behind [3]. APs may be crucial for persons with disabilities, frailty, and chronic diseases, for people with mental health problems, and for ageing people experiencing cognitive and physical decline [3], by supporting people in living healthily, productively, independently and with dignity, enabling participation in education, labor and civic spheres [4]. 

AT provision in many countries, however, particularly low- and middle-income countries, has traditionally been conducted through small-scale local providers, manufacturing products of varying degrees of quality and frequently at a limited price range. An effective way to scale these production and provision enterprises to the required level is needed to close the gap between available and required AT, which is affordable and of acceptable quality. We argue that closing this gap and providing better access to AT will only be achieved through the adoption of a far stronger systems thinking and market shaping approach. Based on a rapid literature review, we present an emergent framework for conceptualizing intersections between systems thinking and market shaping for AT and related technologies—the SMART (Systems-Market for Assistive and Related Technologies) Thinking Matrix. We also consider several challenges for market shaping for AT and related technologies. 

### 1.2. What is Systems Thinking?

Systems thinking has been described as a “discipline for seeing wholes … a framework for seeing inter-relationships rather than things, for seeing patterns of change rather than static ‘snapshots’” [5] (p. 68). A system is composed of “combinations of interrelated, interdependent, or interacting elements forming collective entities” [6] (p. 675). Systems can be either “open” or “closed”. Closed systems operate independently of their environment to produce the same outcome consistently. Open systems, being embedded in their environment, are complex and need to be adaptive or responsive to their environment, and to not necessarily be consistent, but rather to change over time. Closed systems are more characteristic of engineering perspectives, and open systems of social science and service sectors that operate in greatly varied environments [7]. Regarding assistive and related technologies, we need closed systems at the component or product level, where specific technologies reliably produce the same outcomes; and open systems for service provision, which reliably and intelligently adapts to its wider environment and changing demands.

One way of understanding complex, open systems is through Actor-Network Theory (ANT), which has been spearheaded by Latour [8,9] and others who study the “symbiotic” organization of society between human and non-human actors. ANT has helped to illuminate reasons for technology failure, when deployed in complex systems such as transportation; showing how a lack of understanding of the full spectrum of actors has for example led to failure of rapid transport systems in Paris [8,9]. However, frequently, ANT focuses on the agency of a single actor and can become overly focused on the technology or the creator of the technology [10]. Such a focus has received criticism more broadly [11,12] as only looking at and being centered on those who are already within a system; developing a discourse whereby the focus is on “scientists and science” rather than “humans and humanity” [10]. 

Systems thinking has emerged alongside ANT within the social sciences and has also evolved within engineering, healthcare, transport, and other sectors where there is a need to ensure the humanity of a system perseveres within a complex array of variables. To this end, systems theory now has several distinctive features, such as “Forest Thinking”, meaning that there is value in focusing on the wider forest and not only the individual trees; “Dynamic Thinking” recognizes that behaviors are arranged in patterns—not as singular events—that change over time or in different contexts; “Loop Thinking” sees cause and effect as not one-off events and often bi-directional, working in a continuous loop; “System-as-Cause-Thinking” signifies that changes to one aspect of a system can have identifiable effects on other—not directly connected—aspects of the system [13,14]. Systems thinking cuts across disciplinary boundaries. Seeing a system as a *Gestalt*—as the whole being greater than the sum of its parts—and identifying the gaps or blockages within it, is another aspect of systems thinking. Understanding an open system as a whole also requires recognizing that it may be influenced by or permeable to factors beyond that which impinge directly upon the system. Thus, open systems’ boundaries—sometimes also construed as complex adaptive systems—are permeable; and responsive to changes in other systems [15,16], such as the state of the economy, policy and regulatory changes in other sectors, professional protectionism, taxation, and multinationals. Importantly, systems thinking can facilitate interventions at different points or at different levels in the causal network, which contribute to a common overall goal. Systems thinking has recently been applied to the AT sector [7], whereby the authors argue that application of open systems analysis and thinking to the provision and use of AT would lead to “more equitable, more resilient and more sustainable assistive technology across high-, middle- and low-income contexts and countries” [7] (p. 492).

### 1.3. What is Market Shaping? 

Markets for health and welfare products and services can break down when there are incongruities between demand, supply, quality, affordability, and the relationship between public and private sector procurers, producers, and suppliers. Insufficient demand and low-income base can cause investors to view a market as not viable and therefore fail to enter a new area or withdraw from the market. Too much demand with too few suppliers can lead to shortfalls and reduction in quality and reliability. This “market trap” can be overcome through market shaping, which reworks the relationship between the elements of a market in order to strategically ensure that an optimal balance can be achieved, and the availability of higher quality products can be ensured for those who need them, when they need them. Market shaping recognizes that the state of the health and social care system is directly connected to the effectiveness and reach of the markets on which it depends.

The approach to achieving a healthy marketplace is the establishment of strategic relationships between public and private entities, through for example pooled procurement or development and distribution risk sharing, at regional, national, and international levels. By pooling resources and purchasing healthcare products through a non-profit organization, such as GAVI (The Global Alliance for Vaccines and Immunizations) or UNICEF (United Nations International Children’s Emergency Fund), governments can ensure a reduced cost of product, facilitating a more timely agreement with manufacturers, who may then invest in larger-scale production [17]. Such funding structures recognize that solutions require more than a single party’s resources; and individual country preferences may also need to give way to regionally averaged preferences to ensure sufficient product demand that allows for economies of scale [18]. Such public and private strategic arrangements try to avoid the extremes of overly privatized or centralized health systems, through market management (shaping) based on cooperation and partnership [19]. Health system resilience is therefore also linked to the idea of market adaptability.

### 1.4. What is Assistive Technology? 

An AP may be defined as “any product (including devices, equipment, instruments, and software), either especially designed and produced or generally available, whose primary purpose is to maintain or improve an individual’s functioning and independence and thereby promote their well-being” [20] (p. 2229) (see also [21,22]). Common examples of APs include orthotic supporting devices worn on the body, prosthetic arms or legs, wheelchairs or tricycles for people with mobility impairments, screen magnifiers or white canes for people with visual impairments, cochlear implants or hearing aids for people with hearing impairments, communication boards or speech synthesizers for people with speech impairments, and symbol pictures or calendar pill boxes for those with cognitive impairments [20]. 

APs have traditionally been designed for and deployed within a healthcare system. However, increasingly, the boundary between where an AP is designed and provided is being blurred. For instance, Augmentative and Alternative Communication (AAC) has been delivered through trained occupational health specialists and speech and language specialists. However, in recent years, many persons with disabilities needing AACs have been able to switch to, for example, using smartphones. A further disruption to the marketplace includes users of APs being able to tweak open source designs and make their own APs through the global network of market places [23]. This disruption to the way in which APs are designed and distributed, and therefore to the system of AP provision, may include new elements/actors or changing of the roles of established elements/actors within a system [23]. 

The term “assistive technology” is often used generically to refer to the range of technologies available. An AT system refers to “the development and application of organized knowledge, skills, procedures, and policies relevant to the provision, use, and assessment of assistive products” [20] (p. 2229). The “system” in this context may therefore also include other infrastructures and technologies; such as information and communications technologies (ICT), the Internet of Things (IoT), or technologies to promote Ambient Assisted Living (AAL) solutions. These may include ubiquitous computing and sensing technologies, ubiquitous communication, and intelligent user interfaces. Many of these technologies—which may intrinsically be closed systems—operate in an open and complex human system and are therefore often dependent on such a system for their potential benefit to users to actually be realized.

The aim of AT is to help to bridge the gap that exists between a person’s capabilities and what they need to be able to do to achieve their desired goals. When this is achieved, the agency and participation of the person should be improved [24]. If agency and participation are fundamental aspects of development as “the ends and the means” (p. 35) of development interventions [25], then enabling persons with disabilities to participate in society—to speak about their own lives, to shape their families, their communities, the projects that seek to support them, their governments, and research about “them”—is a necessary factor in any development objective. Therefore, co-design of AT can potentially be viewed as more than just a chance to design better APs, but rather it can be viewed as a chance to bring marginalized people together to further their “parity of participation” (p. 115) [26]. Co-production, in this sense, is then viewed as a social development outcome in itself.

### 1.5. Why does Assistive Technology Need Systems Thinking and Market Shaping?

The World Health Organization (WHO) estimates that approximately only 1 in 10 of a billion people who could benefit from APs actually have access to such technologies [27]. Since AT is crucial for the achievement of the SDGs—and to ensuring that nobody is left behind—access to quality and affordable APs is a priority for equitable and inclusive development [3]. The WHO established the Global Cooperation on Assistive Technology (GATE) [28] to address this need. GATE has in turn created a priority assistive products list (APL) [29], indicating 50 APs that should be available in all countries, as a minimum requirement (not a maximum). However, rather than being prescriptive, this list is indicative of priority APs, recognizing that the exact list may vary depending on country context, resources and available systems and expertise.

The history of AT provision in many countries, especially low- and middle-income countries, is one of small-scale local providers manufacturing products of varying degrees of quality and often at a restricted price range. To date, it has not been possible to envisage an effective way of scaling these production and provision enterprises to the level required to close the gap between available and required AT, which is both affordable and of acceptable quality. 

**Figure 1:**
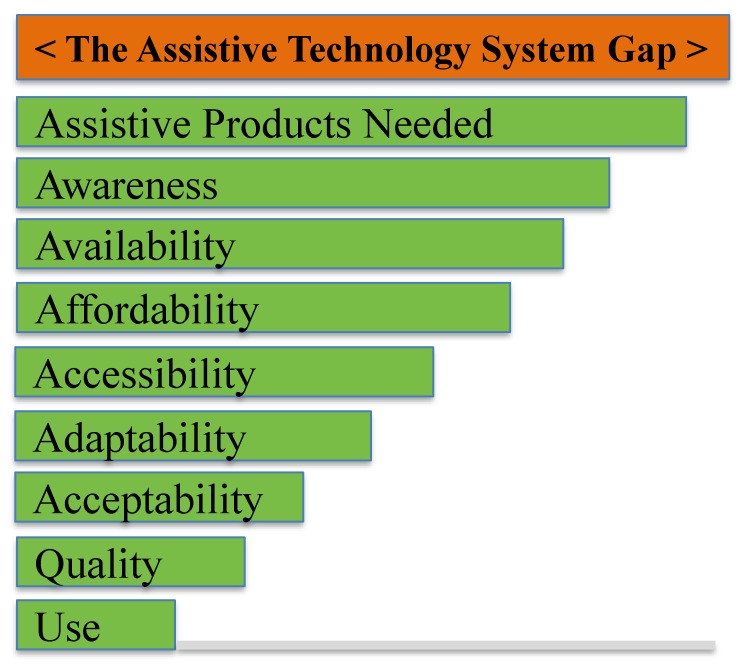
A schematic representation of the Assistive Technology System gap. Note: This bar chart is not to scale—globally the number of APs needed far exceeds those available, sometimes by a ratio of 10 or more to one, and this is patterned by socioeconomic factors, marginalization and so on. Reproduced with permission from MacLachlan et al. [30].

Figure 1 illustrates schematically this AT system gap. It is clear that much of the difference between the APs needed and those used in any particular country may be accounted for, at least to some extent, by a range of factors that are consistent with a market shaping approach. These can be divided into both supply and demand elements. 

On the supply side, quality standards can be created and adopted by Nation States; and on the demand side, work to reduce stigma and increase awareness can be implemented to ensure AT provision will be taken up. Thus, shaping incentives—whether in a social services or commercial market—may contribute to addressing this gap. This requires a systems thinking approach to better leverage market shaping to increase access to AT. As specified by MacLachlan [31], “market shaping means understanding the broader social context of the market, working cooperatively with other stakeholders, and exploring different types of systems to suit different contexts. This requires a degree of systems thinking”. The potential to shape the market towards greater social inclusion and greater supply to those with weak or little purchasing power is of particular interest for the purpose of this analysis. 

## 2. Methods

As market shaping has been used with some success to promote social inclusion in healthcare, including the pharmaceutical sector, we sought to explore its potential within the assistive and related technologies sector. We therefore undertook a focused review targeted on the interplay between AT and relevant market shaping themes. Our aim was to establish a framework for conceptualizing how these two approaches could be understood to interact with each other and identify literature relevant to different forms of such an interaction. Our analysis of the literature emerging from our review therefore sought to establish the extent to which existing literature was relevant to these different types of interaction, and, in so doing, lend some validity to a possible systems level X market functioning framework. 

A flexible literature search was conducted exploring literature relevant to the interface between AT and market shaping. The research focus therefore comprised AT and market shaping. Accordingly, a “scoping study” or rapid review was conducted to explore the range and nature of the research domain and to map the area of study [32]. The search identified 112 texts, outlined in Supplementary Materials.

A flexible search strategy was used, with search terms including “market shaping”, “demand”, “supply”, “fragmentation”; and “assistive technology/technologies”, “assistive device(s)”, and “assistive product(s)”. The search was conducted in English; and no restriction on publication year was specified. Databases and journals were selected as those most relevant to AT and market shaping, with searches conducted on sources including PubMed, National Library of Medicine, Journal STORage (JSTOR), Taylor and Francis Online, Emerald Insight, Stella Search, SpringerLink, Applied Social Sciences Index and Abstracts (ASSIA), ScienceDirect, WHOLIS, EMBASE, SCOPUS, CIRRIE, REHABDATA, LILACS, SCIE, AIM (African Index Medicus), Assistive Technology Journal, Journal of Assistive, Rehabilitative and Therapeutic Technologies, Journal of Enabling Technologies, Technology and Disability Journal, and Disability and Rehabilitation: Assistive Technology. Snowballing comprised searching references of numerous included articles. 

## 3. Results

**Figure 2:**
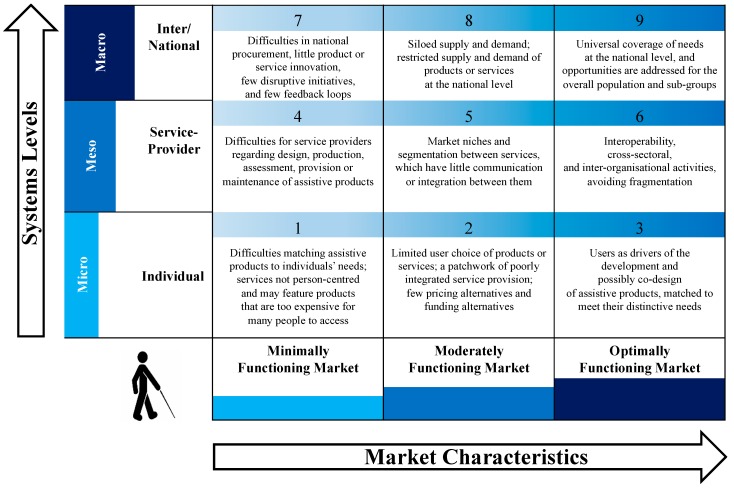
The SMART Thinking Matrix: A matrix showing intersections between systems levels and market characteristics for assistive and related technologies; as supported by research evidence cited in Supplementary Materials.

Based on this literature search, the SMART Thinking Matrix was formulated. The SMART Thinking Matrix depicts the relationship between systems levels and market characteristics within the domain of assistive and related technologies (see Figure 2). The matrix is intended to help people reflect on where they are, either as a user, service provider or policy-maker; or a manufacturer, procurer, or supplier of APs. Boxes 1 and 9 represent the very significant, but quite different, challenges at each of these levels, and it is important to retain this difference between individual and population level needs, ensuring that both are fully addressed. We also believe that the relevance of the matrix may be greater for some APs than for others. 

Systems function at and across macro, meso, and micro levels [33], as depicted in the vertical axis in Figure 2. While in reality the distinction between these levels is often not clear-cut, and more likely a continuum, this categorization is used for illustrative purposes. The macro level focuses primarily at the national or international level. This may be affected by national agreements, laws, or conventional practices; international trade agreements, or United Nations declarations, for instance. It recognizes that the way the system operates at local level is impacted by the broader national and international context. The meso level primarily focuses on how provision is organized for groups—beyond the individual—by service provider organizations, which may operate at a more regional, municipality or local level. The meso level falls between challenges at the individual user/technology level and challenges at the population of users/technologies level. At the meso level, the market in terms of demand and supply may operate in a manner that is not inclusive of all needs or all technologies, as the demand for them or supply chains to them are uncertain or uneconomic. Constricted markets and smaller service providers constitute a challenge to both the individual acquiring APs that they require, and to universal access to such products at the population level. The micro level pertains to individuals and their experience of the system; the extent to which it is user-centred and meets their own distinctive needs. 

The horizontal axis in Figure 2 depicts three levels of market functioning: minimal (where there are significant difficulties at all levels and many potential technology users will not have access to appropriate, or sometimes any, technology products); moderate (where there is access for some people to some products and services, but coverage is selective, not comprehensive or integrated); and optimal (where services and products are user-centred, operate in an integrated manner and provide universal coverage).

At the individual level of the system, Box 1 describes the situation where an individual receives a particular AP(s). While there should be a concern that the product matches the particular needs of the individual—that the technology fits the person rather than the person being fitted into the technology—in a minimally functioning market, this will rarely be the case, except perhaps for wealthy or otherwise well-placed individuals. The individual and her or his advisor/clinicians must choose from what is available, including available support, training, and maintenance—and in many cases, some or all of these may be absent. 

In Box 2, the individual constitutes a single unit within a wider market of people who need APs and have the means to constitute some economic demand. The individual likely has access to a limited variety of products, along with a limited range of prices and funding alternatives (out-of-pocket expenses, personal welfare budgets or public services). It may well be that the service needs are not actually addressed adequately by the vagaries of the market—it may not be profitable for manufacturers to produce specific technologies required by the individual at a price that either the individual or service provider can afford. Furthermore, there may not be appropriate professional expertise available for the specific technology required, particularly where this technology is for addressing impairments of relatively low prevalence.

In Box 3, the market more explicitly works for and with the individual. For instance, the market is providing mechanisms for AT users to co-design the products that they need, tailoring those products to their individual requirements, and doing so as part of an overall system designed to cater both for common high prevalence needs and less common low prevalence needs.

The next system level focuses on the service provider, which may be a community-based organization providing services for hundreds of clients, or a larger organization with thousands of clients. Box 4 describes the situation where there are difficulties for service providers in establishing or maintaining the coherence of services, often resulting in poor reliability and/or quality of service. 

Box 5 describes a situation that is quite common in many countries; where service providers segment the market (for instance, focusing on younger or older users, people with mobility or hearing problems, or technologies for the workplace, or for the home). While the identification of these niches is not in itself problematic, what becomes difficult for the user is when there is little or no communication between these different segments, and the user must traverse the relationships, inconsistencies and sometimes incompatibilities. For instance, an older service user with an intellectual disability and poor vision may wish to use the same screen reader at home and in their workplace, with these however only being provided for younger persons at special schools. 

In Box 6, while specialization for different cohorts of people or different types of technologies may occur, the emphasis is on the service and the market interacting to promote interoperability, communicating across sectors and preventing fragmentation of services; thus minimizing the onus and burden on the user.

As outlined above, the macro level concerns international or national population-wide systems. These may draw on many entities within the country, but increasingly also on regulations, products, or personnel from elsewhere; as the AT sector becomes increasingly global. Box 7 illustrates that there may be a small range of products provided through national-level procurement; there may be little product or service innovation; and few examples of constructive disruption of existing inadequate practices. There may also be a lack of feedback from product user to product developer or product procurer at the national level.

Box 8 describes the situation that is also common in many countries, where certain types of technology may be well supplied to certain groups, but other groups that may have fewer people in them, be less vocal, less effective advocates, or have less political clout, do not have access to the sorts of products that they need. Furthermore, manufacturers and suppliers may prefer to target only those products that produce consistently profitable returns, in mature reliable markets. It may well be that there are some specific groups, or specialized technologies, where it is simply not worth the time or financial investment for producers or suppliers to get involved.

In Box 9, the macro level system interacts with the ethos of market shaping, seeking to ensure that all those who require any type of AP have access to a quality product of the required type and at an affordable price. This may be achieved through a combination of market shaping techniques as described earlier, such as pooling procurement or underwriting market demand, to encourage producers to enter the market.

## 4. Discussion

We next consider three particular challenges for market shaping in the context of assistive and related technologies:

1. How to organize to achieve optimal market shaping?
2. What regulatory issues may be related to market shaping?
3. How might market shaping happen in contexts where procurement-to-scale of AT may be dominated by powerful external stakeholders?

### 4.1. Organizing Systems Thinking and Market Shaping 

In line with an ecosystems approach to market shaping that recognizes the need for adaptability and scalability, there is a requirement for cooperative planning incorporating the voices of various actors and stakeholders (service users, providers, suppliers, procurers, manufacturers, designers, researchers, regulators etc.). By combining the methodology of the Concept of Operations or CONOPS approach [34] and the activity systems thinking approach of Cultural Historical Activity Theory [35,36], we can start to develop an integrated framework of system actors orientated towards goals that are simultaneously individual and collective (personal and systemic), mediated by tools, including technologies and methods/techniques, and situated within a system of relationships between people and organizations. 

A CONOPS is a user-orientated document or representation that describes the functioning of a proposed (future) system from the perspective of the actors within it [37], including how they interact with tools and other resources, and how their activities intersect. As a method for supporting design, CONOPS has a history in the software development community and uses an IEEE (Institute of Electrical and Electronics Engineers) standard framework [37]. CONOPS is also used in crisis and emergency management for strategic and tactical planning of operations to ensure the outcomes are understood by all actors including how activities intersect. If the “spirit” of a plan and its objectives are internalized, this can help localized actors self-organize to achieve goals consistent with the overall objective without requiring micromanagement and allowing local independence. CONOPS development needs to be agile [38] and reflect a multi-voiced integrated system vision.

Cultural Historical Activity Theory complements the CONOPS approach by seeing the structure of a system as a structure of relationships that are dynamic in nature and essentially social. Activities of individuals, groups and organizations are orientated towards socially meaningful objectives or goals. One of the strengths of this approach is the acknowledgement of actual and potential contradictions (conflicts) between elements within and between systems. For example, the need an individual may have for an AT capability may be at odds with the supply, quality, or cost of that resource. This helps us to orientate ourselves critically but constructively towards identifying the factors that shape a successful AT market, and shape solutions towards achieving that through dialogue and a commitment towards a shared vision of a system based on societal values. To move from an existing non-ideal system to a system that can address individual, organizational and population level needs requires developing and executing a “Theory of Change” with a broad range of stakeholders, which can influence policy and systems development [30].

This historical dimension of Activity Theory is also a crucial consideration as it relates to the notion of path dependency [39], which states that our current framework of tools, objects and methods is a result of previous structural decisions which, over time, may have a degree of inertia built in whereby the costs of change, or perceived changes, outweigh any immediate gain, at least in the short-term. This is a key challenge for market shaping. We cannot expect that significant systemic changes in the relationships between producer, vendor and supplier can be achieved overnight. It amounts to a type of prisoner’s dilemma problem, discussed by Grudin [40]. While Activity Theory does not offer an immediate solution to this issue, it does require us to take an historical perspective on not only the relationship between the past and present but also the future. By taking a planned, object-orientated approach to deciding collaboratively where we want to be as both a society and as a market, and how to get there, we can ease ourselves into the achievement of reducing the dependency on existing pathways and provide the scope for innovation to catch on successfully.

### 4.2. Legal Issues Intersecting Systems Thinking and Market Shaping

National, supranational (e.g. European Union), and international laws may play a role in market shaping. The horizontal and vertical (multi-level) interaction between different sources of law, at the macro level, as described above, can affect the production, supply and/or delivery of AT. For this analysis, three basic ways in which the law “intersects” the macro level can be identified.

First, international law interacts with the ethos of market shaping because it can nudge States towards ensuring that all those who require any type of AP have access to it. This is, for example, the case of the United Nations Convention on the Rights of Persons with Disabilities (CRPD) [41], which places great emphasis on APs. Article 4(1)(g) of the CRPD obliges States Parties to “undertake or promote research and development of, and to promote the availability and use of new technologies, including ICT, mobility aids, devices and assistive technologies, suitable for persons with disabilities, giving priority to technologies at an affordable cost”. Article 4(1)(h) of the CRPD requires States Parties to provide “accessible information to persons with disabilities about mobility aids, devices and assistive technologies, including new technologies”. Article 9 of the CRPD imposes a range of duties on States Parties to the Convention, making it clear that accessibility covers more than technical design specifications for products, information and signage, or the built environment. It also establishes that technologies must be accessible, and explicitly requires that ICT are made accessible to people with disabilities at a minimum cost. Article 20 of the CRPD, on personal mobility, imposes on States Parties the obligations to facilitate access “to quality mobility aids, devices, assistive technologies and forms of live assistance and intermediaries, including by making them available at affordable cost”. Other provisions include references to APs. In addition, Article 32 of the CRPD, on international cooperation, requires States Parties *inter alia* to facilitate access to and sharing of accessible and assistive technologies.

Second, various regulations at different levels can influence the production of APs and constrain the behaviors of market operators locally and globally (the term “regulation” is used as a general umbrella term; and is different from and broader than “legislation”. It includes legislation, bylaws, and soft law. It also includes “private regulation”, for example, regulatory contracts, codes of conduct and voluntary agreements by which economic actors, social players, non-governmental organizations and organized groups establish themselves to voluntarily regulate and organize their activities). For example, international and supranational law can regulate the role of the State in supporting economic operators and influence the choices of manufacturers, especially in the case of products that do not produce profitable returns. The World Trade Organization Agreement on Subsidies and Countervailing Measures places definite constraints on the possibility for States to subsidize national industries. In the European Union (and in neighbouring countries), subsidies are, in principle, prohibited. It is well known that Article 107(1) of the Treaty on the Functioning of the European Union (TFEU) provides that any aid granted by a Member State or through State resources, which distorts or threatens to distort competition by favoring certain undertakings or the production of certain goods, is incompatible with the internal market, insofar as it affects trade between the Member States. However, Article 107(2) and (3) of the TFEU set out derogations to the general ban contained in Article 107(1) of the TFEU on the premise that markets do not always self-regulate effectively, and State intervention may be required for this purpose and to raise consumer welfare or protect and promote specific rights or values. These derogations have left the door open to national subsidies or de-taxation measures to boost the production of APs [42,43]. National and EU competition law, which ensure the existence of a competitive market by regulating (and prohibiting) anti-competitive conduct by companies, can also deeply influence the production of APs. In fact, they directly affect the behavior of market operators by outlawing anti-competitive arrangements or abuse of dominance, but also by controlling mergers, acquisitions, and joint ventures. 

Third, in relation to the supply and delivery of APs, national and EU public procurement law might influence the way in which APs are supplied or delivered through public services. At present, most APs are provided through health or social services, or, more generally, support schemes for people with disabilities. However, regulations on the provision of APs can also vary according to the context (e.g. educational or workplace) or type of product [44].

### 4.3. Market Shaping in Contexts Where Procurement-to-Scale of AT May Be Dominated by Powerful External Stakeholders

In higher-income contexts, there may be a range of competing manufacturers, suppliers, and providers, all of which may be able to work to the scale and standards required at the national level. In lower-income contexts, however, there may be a lack of resources or expertise to achieve the scale required to ensure increased production to allow access to the required level of APs. Clearly, one response to this is investment in local production capabilities, as well as the system to implement equitable provision of AT. In the short run, and perhaps in conjunction with existing local suppliers, we should explore the possibility of procuring large numbers of quality and affordable APs that could be distributed globally.

It may therefore be opportune to engage producers elsewhere that have the potential of providing quality affordable products at a scale not hitherto achieved. However, while such procurement could have considerable benefits in terms of security of supply chain, quality of products and supporting maintenance, it also runs the risk of undermining social entrepreneurship in this area, which has been characteristic of local small-scale production in resource constrained contexts. It is, therefore, important to identify ways in which these community-based skills can be combined with the security of production and supply that may be more likely provided by stakeholders operating at a global level. 

One possible configuration for the sort of relationship described above is where AT components are procured nationally, while at local community level entrepreneurship is encouraged through both the skillful assembly of AT components, their customization relative to the needs of individual users, and the development of a variety of service delivery and support options [45]. Building on the lessons learnt from market shaping in the context of viruses, it will be important to ensure end-to-end planning in terms of provision, demands, information, maintenance, and end-user feedback into a system that is capable of correcting and shaping the market according to basic needs for quality and affordable AT [19].

There are many elements of AT services that could provide scope for innovative packaging of AT services at community level, embracing local entrepreneurship, overall product enhancement and potentially greater user satisfaction. These include:

1. Assessment of the need for particular APs.
2. Assembly of subcomponents of specific products at the community level.
3. Customization of products in terms of aesthetic preferences.
4. Fitting of products.
5. Support, maintenance and follow-up of products and users.
6. Provision of different types of servicing agreements.
7. Inclusion of product users through co-design.
8. Guarantees on parts and/or servicing.
9. Auxiliary services—such as cleaning, decorating, providing an alternative when the usual product is being repaired, or co-location with other services that might be relevant to product users (for instance physiotherapy, employment, or social welfare services).

There are many examples of innovation in other service industries that use similar materials. For instance, there are similar raw materials provided and strong competition in the servicing or retail sectors. For example, petrol or coffee are provided through very different outlet models, while the basic material is almost homogenous. In many countries, enterprising individuals have set up services assembling IKEA furniture and compete in terms of price and the nature of the service offered. Here the basic materials are the same. In the banking industry, mortgage lenders produce hugely varied products for the same basic service—borrowing money. Thus, there are many examples where the retail or service provision aspect of the supply chain can create a lively, profitable, and meaningful degree of market segmentation. 

## 5. Future Research Recommendations

The SMART Thinking Matrix should stimulate both reflections on policy and practice, as well as ideas for future research. Research recommendations include:

1. What are the barriers and facilitators to adopting a more systemic approach to assistive and related technologies?
2. Which government departments should be central to a systemic approach to assistive and related technologies?
3. How can systems thinking be used to stimulate innovation in assistive and related technologies?
4. What are the barriers and facilitators to moving from minimally to moderately to optimally functioning markets for assistive and related technologies?
5. How can strategic alliances be formed between private, public, and voluntary sectors to ensure access to quality and affordable assistive and related technologies, for all?
6. How can product or service innovation for assistive and related technologies be most effectively stimulated?
7. How can the primacy of the product and service user be incorporated in national-level planning for assistive and related technologies?
8. How can key contextual factors (resources, infrastructure, and culture for example) be taken into account to promote the coherence of access to assistive and related technologies, in different countries?

These research questions are simply illustrative of how the matrix can stimulate important and relevant avenues for research. 

## 6. Conclusions

The aim of this study was to explore intersections between systems thinking and market shaping for assistive and related technologies, and to develop a framework for conceptualizing such interactions. A focused review was undertaken to establish a framework for conceptualizing how AT and market shaping could be understood to interact with each other and identify literature relevant to different forms of such an interaction. The SMART Thinking Matrix was therefore formulated, which describes the relationship between systems levels and market characteristics for assistive and related technologies. 

States Parties to the CRPD have undertaken to “promote research and development of, and to promote the availability and use of new technologies, including information and communications technologies, mobility aids, devices and assistive technologies” [41] (Article 4). Fair access to AT is crucial on the grounds of equity, social justice, and rights [46], and for the equitable realization of the SDGs [3]. However, fair access to AT will only be realized through the adoption of a far stronger market shaping and systems thinking approach [46]. Indeed, market shaping is primarily needed when market economics fail to facilitate social gain in a fair and equitable way [45]. A stronger systems thinking approach in the AT sector will also enable more equitable, resilient, and sustainable AT provision in low-, middle- and high-income contexts [7,30]. By using a systems thinking approach to better leverage market shaping, there is considerable potential to develop a stronger, fairer, and more resilient market for AT in poorly resourced settings. This is one of the aims of AT 2030 [47], of which several of the authors of this paper are associated. 

Many AT services, particularly in resource poor countries or regions, have only become possible through the development of meso level provision, through a restricted number and scope of service providers. Such services have been crucial in giving access to APs where none may have previously existed. However, in line with the SDGs and their aspiration to “leave no-one behind”, it is increasingly important to think of both product and service provision at the systems and market shaping level; and how this can contribute to the challenge of scaling the production and provision of APs, so that the 90% of those who need them, but do not have access to them, can benefit from quality, affordable assistive products and related technologies.

## Supplementary Materials

The following are available online at http://www.mdpi.com/1660-4601/15/12/2627/s1, Figure S1: The SMART Thinking Matrix.
