# Supplementary material for: Intersections Between Systems Thinking and Market Shaping for Assistive Technology: The SMART (Systems-Market for Assistive and Related Technologies) Thinking Matrix
Source: Int J Environ Res Public Health. 2018 Nov 23;15(12):2627. doi: 10.3390/ijerph15122627 (PMC6313499; doi:10.3390/ijerph15122627)
Supplement: Supplementary file 1 [file ijerph-15-02627-s001.pdf]

International Journal of
Environmental Research and Public Health

MDPI

Supplementary Figure S1. The SMART Thinking Matrix

Systems Levels	**Inter/National** *Macro*	**7** 27,34,46,51,54,55,58,59,61,62, 63,65,67,68,72,74,76,81,82,83, 84,88,90,94,99,104,105,108	**8** 6,8,11,15,16,17,18,21, 24,28,29,31,32,55,65, 66,70,71,104,106,111	**9** 4,7,9,10,14,20,22,25, 26,30,57,63,64,65,69, 87,108
	**Service-Provider** *Meso*	**4** 23,35,41,45,51,56,58,59,66,70, 80,91,96,98,101,105,110,111	**5** 14,63,75,78,79,82,85, 86,88,93,95	**6** 5,37,38,40,49,51,53, 54,62,65,69,89,93,97, 105,106,109,110,112
	**Individual** *Micro*	**1** 1,3,12,19,31,42,44,45,47,48, 55,60,62,77,80,85,92,100,102, 103,105,106,112	**2** 1,2,44,50,52,53,54,55, 65,77,86,91,92,107	**3** 13,33,36,39,43,73,85, 97,105
		**Minimally Functioning Market**	**Moderately Functioning Market**	**Optimally Functioning Market**
		**Market Characteristics**		

Note: The SMART Thinking Matrix: A matrix showing intersections between systems levels and market characteristics for assistive and related technologies; as supported by research evidence cited below.

References from Literature Search in relation to the SMART Thinking Matrix:

1. Andrich, R., & Caracciolo, A. (2007). Analysing the cost of individual assistive technology programmes. *Disability and Rehabilitation: Assistive Technology*, *2*(4), 207-234. DOI: 10.1080/17483100701325035
2. Harris, F., & Sprigle, S. (2003). Cost analyses in assistive technology research. *Assistive Technology*, *15*(1), 16-27. DOI: 10.1080/10400435.2003.10131886
3. Scherer, M. J., & Craddock, G. (2002). Matching person & technology (MPT) assessment process. *Technology & Disability, Special Issue: The Assessment of Assistive Technology Outcomes, Effects and Costs*, *14*(3), 125-131.
4. Lane, J. P. (1997). Roles for the technology transfer intermediary. In G. Anogianakis, C. Buhler, & M. Soede (Eds.), *Advancement of Assistive Technology* (pp. 357-362). Amsterdam, Netherlands: IOS Press.
5. Battistella, L. R., Juca, S. S. H., Tateishi, M., Oshiro, M. S., Inglez Yamanaka, E., Lima, E., & Delgado Ramos, V. (2015). Lucy Montoro Rehabilitation Network mobile unit: An alternative public healthcare policy. *Disability and Rehabilitation: Assistive Technology*, *10*(4), 309-315. DOI: 10.3109/17483107.2015.1027294
6. Bauer, S. M., Elsaesser, L.-J., & Arthanat, S. (2011). Assistive technology device classification based upon the World Health Organization's International Classification of Functioning, Disability and Health (ICF). *Disability and Rehabilitation: Assistive Technology*, *6*(3), 243-259. DOI: 10.3109/17483107.2010.529631
7. Borg, J., Lindstrom, A., & Larsson, S. (2011). Assistive technology in developing countries: A review from the perspective of the Convention on the Rights of Persons with Disabilities. *Prosthetics and Orthotics International*, *35*(1), 20-29. DOI: 10.1177/0309364610389351
8. Buhler, C., & Barbera, R. (2011). Assistive technology industry: A field for cooperation and networking. *Technology and Disability*, *23*, 115-130. DOI: 10.3233/TAD-2011-0320
9. Consolidating Logistics for Assistive Technology Supply and Provision (CLASP).

10. *About CLASP: Consolidating logistics for assistive technology supply and provision.* Retrieved from https://www.clasphub.org/about-us/
11. Van der Peijl, S., Munisteri, F., Negreiro, M., Kapff, L., Jermolina, V., & Folkes, C. (2011). *The internal market for assistive ICT: Executive summary of the final report.* Deloitte, and AbilityNet.
12. Harniss, M., Raja, D. S., & Matter, R. (2015). Assistive technology access and service delivery in resource-limited environments: Introduction to a special issue of Disability and Rehabilitation: Assistive Technology. *Disability & Rehabilitation: Assistive Technology*, *10*(4), 267-270. DOI: 10.3109/17483107.2015.1039607
13. Johnston, P., Currie, L. M., Drynan, D., Stainton, T., & Jongbloed, L. (2014). Getting it “right”: How collaborative relationships between people with disabilities and professionals can lead to the acquisition of needed assistive technology. *Disability and Rehabilitation: Assistive Technology*, *9*(5), 421-431. DOI: 10.3109/17483107.2014.900574
14. Kylberg, M., Löfqvist, C., Tomsone, S., Phillips, J., Liepina, Z., & Iwarsson, S. (2015). A European perspective on the service delivery systems for assistive technology – Differences and similarities between Latvia and Sweden. *Journal of Cross-Cultural Gerontology*, *30*(1), 51-67. DOI: 10.1007/s10823-014-9255-4
15. Lane, J. P. (2003). The state of the science in technology transfer: Implications for the field of assistive technology. *Journal of Technology Transfer*, *28*(3-4), 333-354.
16. Marasinghe, K. M., Lapitan, J. M., & Ross, A. (2015). Assistive technologies for ageing populations in six low-income and middle-income countries: A systematic review. *BMJ Innovations*, *1*(4), 182-195. DOI: 10.1136/bmjinnov-2015-000065
17. Mayer, P., Hauer, K., Schloffer, E., & Leyrer, B. (2015). Assistive technologies along supply chains in health care and in the social services sector. *Health Informatics Meets E-health.* DOI: 10.3233/978-1-61499-524-1-111
18. McPherson, B. (2014). Hearing assistive technologies in developing countries: Background, achievements and challenges. *Disability and Rehabilitation: Assistive Technology*, *9*(5), 360-364. DOI: 10.3109/17483107.2014.907365
19. Pearlman, J., Cooper, R. A., Zipfel, E., Cooper, R., & McCartney, M. (2006). Towards the development of an effective technology transfer model of wheelchairs to developing countries. *Disability and Rehabilitation: Assistive Technology*, *1*(1-2), 103-110. DOI: 10.1080/09638280500167563
20. Ravneberg, B. (2009). Identity politics by design: Users, markets and the public service provision for assistive technology in Norway. *Scandinavian Journal of Disability Research*, *11*(2), 101-115. DOI: 10.1080/15017410902753904
21. Rice, D. (2015). Public procurement as a means to achieving social gains – progress and challenges in European legislation and standards for accessible information and communication technology. *International Review of Law, Computers & Technology*, *29*(2-3), 162-182. DOI: 10.1080/13600869.2015.1055661
22. Rios, A., Cruz, A. M., Guarin, M. R., & Villarraga, P. S. C. (2014). What factors are associated with the provision of assistive technologies: The Bogatá D. C. case. *Disability and Rehabilitation: Assistive Technology*, *9*(5), 432-444. DOI: 10.3109/17483107.2014.936053
23. Seelman, K. D., & Werner, R. (2014). Technology transfer of hearing aids to low and middle income countries: Policy and market factors. *Disability and Rehabilitation: Assistive Technology*, *9*(5), 399-407. DOI: 10.3109/17483107.2014.905641
24. Saloojee, G., Phohole, M., Saloojee, H., & Ijsselmuiden, C. (2006). Unmet health, welfare and educational needs of disabled children in an impoverished South African peri-urban township. *Child: Care, Health and Development*, *33*(3), 230-235. DOI: 10.1111/j.1365-2214.2006.00645.x
25. Stephanidis, C., Vernardakis, N., & Akoumianakis, D. (1994). The demand, supply and provision system of the rehabilitation technology market in Europe: A modelling perspective. *International Journal of Rehabilitation Research*, *17*(4), 343-56.
26. Spoehr, J., Worrall, L., Molloy, S., Sandercock, P., & Fraunhofer, I. A. O. (2017). *Assisting transition: Growth prospects for the development of the assistive technology industry in South Australia.* Adelaide, Australia: Australian Industrial Transformation Institute, Flinders University of South Australia. Retrieved from http://www.flinders.edu.au/fms/AITI/Documents/AITI201602_Assisting_Transition.pdf
27. Spoehr, J., Worrall, L., Sandercock, P., Eyre, J., & Molloy, S. (2014). *Assisting transition: Assistive technologies opportunities and industrial transformation in South Australia*. Adelaide, Australia: Australian Workplace Innovation and Social Research Centre, The University of Adelaide. Retrieved from http://www.flinders.edu.au/fms/AITI/Documents/assistingtransition-april2014.pdf
28. Summers, M. P., & Verikios, G. (2018). Assistive technology pricing in Australia: Is it efficient and equitable? *Australian Health Review*, *42*, 100-110. DOI: 10.1071/AH16042

29. Tinghog, G., & Carlsson, P. (2012). Individual responsibility for healthcare financing: Application of an analytical framework exploring the suitability of private financing of assistive devices. *Scandinavian Journal of Public Health*, *40*(8), 784-794. DOI: 10.1177/1403494812462459
30. Vernardakis, N., Stephanidis, C., & Akoumianakis, D. (1994). Rehabilitation technology product taxonomy: A conceptual tool for analysing products and extracting demand determinants. *International Journal of Rehabilitation Research*, *17*(3), 201-214.
31. World Health Organization (WHO). (2016). *Priority assistive products list: Improving access to assistive technology for everyone, everywhere.* Geneva, Switzerland: WHO. Retrieved from http://apps.who.int/iris/handle/10665/207694
32. Adya, M., Samant, D., Scherer, M. J., Killeen, M., & Morris, M. W. (2012). Assistive/rehabilitation technology, disability, and service delivery models. *Cognitive Processing*, *13*(1), S75-S78. DOI: 10.1007/s10339-012-0466-8
33. Banes, D. (2007). Delivering assistive technology services through a barrier-free approach: The use of Web 2.0 in the UK. *Journal of Assistive Technologies*, *1*(2), 39-42.
34. Ravneberg, B., & Soderstrom, S. (2017). *Disability, society and assistive technology.* Routledge.
35. Banes, D. (2016). *How disruptive technology is shaping access.* NS Tech. Retrieved from http://tech.newstatesman.com/enterprise-it/disruptive-technology-shaping-assistive
36. World Health Organization (WHO). (2016). *Assistive technology: Fact sheet.* Geneva, Switzerland: WHO. Retrieved from http://www.who.int/mediacentre/factsheets/assistive-technology/en/
37. Ratzka, A. (2003). *From patient to customer: Direct payments for assistive technology for disabled people's self-determination.* Farsta, Sweden: Independent Living Institute. Retrieved from https://www.independentliving.org/docs6/ratzka200308b.html
38. Borg, J., Lindström, A., & Larsson, S. (2009). Assistive technology in developing countries: National and international responsibilities to implement the Convention on the Rights of Persons with Disabilities. *Lancet*, *374*(9704), 1863-65. DOI: 10.1016/S0140-6736(09)61872-9
39. Cullen, K., McAnaney, D., Dolphin, C., Delaney, S., & Stapleton, P. (2012). *Research on the provision of assistive technology in Ireland and other countries to support independent living across the life cycle.* Dublin, Ireland: Work Research Centre. Retrieved from http://nda.ie/File-upload/Research-on-the-provision-of-Assistive-Technology1.pdf
40. Egan, K. J., & Pot, A. M. (2016). Encouraging innovation for assistive health technologies in dementia: Barriers, enablers and next steps to be taken. *Journal of the American Medical Directors Association*, *17*(4), 357-363. DOI: 10.1016/j.jamda.2016.01.010
41. Finn, R. L., & Wright, D. (2011). Mechanisms for stakeholder co-ordination in ICT and ageing. *Journal of Information, Communication and Ethics in Society*, *9*(4), 265-286. DOI: 10.1108/14779961111191066
42. Gould, M., Leblois, A., Bianchi, F. C., & Montenegro, V. (2015). Convention on the Rights of Persons with Disabilities, assistive technology and information and communication technology requirements: Where do we stand on implementation? *Disability and Rehabilitation: Assistive Technology*, *10*(4), 295-300. DOI: 10.3109/17483107.2014.979332
43. Heerkens, Y., Bougie, T., & Claus, E. (2011). The use of the ICF in the process of supplying assistive products: Discussion paper based on the experience using a general Dutch prescription guideline. *Prosthetics and Orthotics International*, *35*(3), 310-317. DOI: 10.1177/0309364611419890
44. Lewis, A. N., Cooper, R. A., Seelman, K. D., Cooper R., & Schein, R. M. (2012). Assistive technology in rehabilitation: Improving impact through policy. *Rehabilitation Education*, *26*(1), 19-32.
45. Martin, J. K., Martin, L. G., Stumbo, N. J., & Morrill, J. H. (2011). The impact of consumer involvement on satisfaction with and use of assistive technology. *Disability and Rehabilitation: Assistive Technology*, *6*(3), 225-242. DOI: 10.3109/17483107.2010.522685
46. Moody, A. K. (2015). Procedures, considerations, and recommendations for the development of an assistive technology demonstration and lending sites. *Journal of Special Education Technology*, *30*(3), 179-187. DOI: 10.1177/0162643415619248
47. Pal, J., Vartak, A., Vyas, V., Chatterjee, S., Paisios, N., & Cherian, R. (2010). A ratification of means: International law and assistive technology in the developing world. ICTD 2010 Proceedings of the 4th ACM/IEEE International Conference on Information and Communication Technologies and Development. DOI: 10.1145/2369220.2369247
48. Ravneberg, B. (2012). Usability and abandonment of assistive technology. *Journal of Assistive Technologies*, *6*(4), 259-269. DOI: 10.1108/17549451211285753

49. Reisinger, K. D., & Ripat, J. D. (2014). Assistive technology provision within the Navajo nation: User and provider perceptions. *Qualitative Health Research*, *24*(11), 1501-1517. DOI: 10.1177/1049732314546755
50. Samant, D., Matter, R., & Harniss, M. (2013). Realizing the potential of accessible ICTs in developing countries. *Disability and Rehabilitation: Assistive Technology*, *8*(1), 11-20. DOI: 10.3109/17483107.2012.669022
51. Schüler, E., Salton, B. P., Sonza, A. P., Façanha, A. R., Cainelli, R., Gatto, J., . . . Araújo, M. C. C. (2013). Production of low cost assistive technology. MEDES 2013, Proceedings of the Fifth International Conference on Management of Emergent Digital EcoSystems, 297-301.
52. Tebbutt, E., Brodmann, R., Borg, J., MacLachlan, M., Khasnabis, C., & Horvath, R. (2016). Assistive products and the Sustainable Development Goals (SDGs). *Globalization and Health*, *12*(79), 1-6. DOI: 10.1186/s12992-016-0220-6
53. Wallace, J. F. (2003). A policy analysis of the assistive technology alternative financing program in the United States. *Journal of Disablity Policy Studies*, *14*(2), 74-81.
54. World Health Organization (WHO). (2010). *Medical devices: Managing the mismatch. An outcome of the priority medical devices project.* Geneva, Switzerland: WHO. Retrieved from http://apps.who.int/iris/bitstream/10665/44407/1/9789241564045_eng.pdf
55. Andrich, R., Mathiassen, N.-E., Hoogerwerf, E.-J., & Gelderblom, G. J. (2013). Service delivery systems for assistive technology in Europe: An AAATE/EASTIN position paper. *Technology and Disability*, *25*(3), 127-146. DOI: 10.3233/TAD-130381
56. Andrich, R. (2016). Re-thinking assistive technology service delivery models in the light of the UN Convention. In K. Miesenberger, C. Bühler, & P. Penaz (Eds.), *Computers helping people with special needs* (pp.101-108). ICCHP 2016. Lecture Notes in Computer Science, 9758. Cham, Switzerland: Springer. DOI: 10.1007/978-3-319-41264-1_13
57. Anttila, H., Samuelsson, K., Salminen, A.-L., & Brandt, Å. (2012). Quality of evidence of assistive technology interventions for people with disability: An overview of systematic reviews. *Technology and Disability*, *24*(1), 9-48.
58. DOI: 10.3233/TAD-2011-0332
59. Caccamo, S., Voloshchenko, A., & Dankyi, N. Y. (2014). The importance of creating a social business to produce low-cost hearing aids. *Disability and Rehabilitation: Assistive Technology*, *9*(5), 368-373. DOI: 10.3109/17483107.2014.906664
60. Carkeet, D., Pither, D., & Anderson, M. (2014). Service, training and outreach – The EARS Inc. model for a self sustainable hearing program in action. *Disability and Rehabilitation: Assistive Technology*, *9*(5), 383-390.
61. DOI: 10.3109/17483107.2014.914979
62. Clark, J. L., & Swanepoel, D. W. (2014). Technology for hearing loss – As We Know it, as We Dream it. *Disability and Rehabilitation: Assistive Technology*, *9*(5), 408-413. DOI: 10.3109/17483107.2014.905642
63. De Gois Pinto, M., Thomann, G., & Villeneune, F. (2016). Assistive products development: A framework to respond to the value requirements from users' and manufacturers' points of view. *Procedia CIRP*, *50*, 559-564. DOI: 10.1016/j.procir.2016.04.203
64. Emiliani, P. L., Stephanidis C., & Vanderheiden, G. (2011). Technology and inclusion – Past, present and foreseeable future. *Technology and Disability*, *23*(3), 101-114. DOI: 10.3233/TAD-2011-0319
65. Federici, S., Corradi, F., Meloni, F., Borsci, S., Mele, M. L., de Sylva, S. D., & Scherer, M. J. (2014). A person-centred assistive technology service delivery model: A framework for device selection and assignment. *Life Span and Disability*, *17*(2), 175-198.
66. Fels, D. I., & Gedeon, S. A. (2011). Understanding motivations of entrepreneurs in the assistive technology market. *Technology and Disability*, *23*(2), 53-64. DOI: 10.3233/TAD-2011-0314
67. Ferri, D., Giannoumis, G. A., & O'Sullivan, C. E. (2015). Fostering accessible technology and sculpting an inclusive market through regulation. *International Review of Law, Computers, and Technology*, *29*(2-3), 81-87. DOI: 10.1080/13600869.2015.1055666
68. Ferri, D. (2015). Does accessible technology need an 'entrepreneurial state'? The creation of an EU market of universally designed and assistive technology through state aid. *International Review of Law, Computers and Technology*, *29*(2-3), 137-161. DOI: 10.1080/13600869.2015.1055660
69. Harkins, C. S., McGarry, A., & Buis, A. (2012). Provision of prosthetic and orthotic services in low-income countries: A review of the literature. *Prosthetics and Orthotics International*, *37*(5), 353-361. DOI: 10.1177/0309364612470963

70. Kohlbacher, F., & Hang, C. C. (2011). Applying the disruptive innovation framework to the silver market. *Ageing International*, *36*(1), 82-101. DOI: 10.1007/s12126-010-9076-x
71. Lane, J. P. (2015). Delivering beneficial impacts in assistive technology: Improving government's approach to innovation. In C. Sik-Lanyi, E.-J. Hoogerwerf, K. Miesenberger, & P. Cudd (Eds.), *Assistive technology: Building bridges* (pp. 78-83). Amsterdam, Netherlands: IOS Press.
72. Lane J. P. (1995). Toward a single global market for assistive technology: U.S. electronic links. In I. P. Porrero, & R. P. de la Bellacasa (Eds.), *The European context for assistive technology, Proceedings of the 2nd TIDE Congress, 26-28 April 1995, Paris* (pp. 143-146). Amsterdam, Netherlands: IOS Press.
73. Matter, R., Harniss, M., Oderud, T., Borg, J., & Eide, A. H. (2017). Assistive technology in resource limited environments: A scoping review. *Disability and Rehabilitation: Assistive Technology*, *12*(2), 105-114. DOI: 10.1080/17483107.2016.1188170
74. Mayer, P., Hauer, K., Schloffer, E., & Leyrer, B. (2015). Assistive technologies along supply chains in health care and in the social services sector. In D. Hayn, G. Schreier, E. Ammenwerth, & A. Horbst (Eds.), *e-health2015 – Health informatics meets ehealth; Innovative health perspectives: Personalized health* (pp. 111-116). Amsterdam, Netherlands: IOS Press.
75. McCarthy, A. D. (2012). Regulatory influences on assistive technology innovation: Enabling or disabling? *Technology and Disability*, *24*(3), 205-210. DOI: 10.3233/TAD-2012-0350
76. O'Rourke, P., Ekins, R., Timmins, B., Timmins, F., Long, S., & Coyle, E. (2014). Crucial design issues for special access technology; A Delphi study. *Disability and Rehabilitation: Assistive Technology*, *9*(1), 48-59. DOI: 10.3109/17483107.2013.806599
77. Pearlman, J., Cooper, R., Chhabra, H. S., & Jefferds, A. (2009). Design, development and testing of a low-cost electric powered wheelchair for India. *Disability and Rehabilitation: Assistive Technology*, *4*(1), 42-57. DOI: 10.1080/17483100802338440
78. Robinson, L., Gibson, G., Kingston, A., Newton, L., Pritchard, G., Finch, T., & Brittain, K. (2013). Assistive technologies in caring for the oldest old: A review of current practice and future directions. *Aging Health*, *9*(4), 365-375.
79. Shin, J., Kim, Y., Nam, H., & Cho, Y. (2016). Economic evaluation of healthcare technology improving the quality of social life: The case of assistive technology for the disabled and elderly. *Applied Economics*, *48*(15), 1361-1371. DOI: 10.1080/00036846.2015.1100254
80. Steel, E. J., Layton, N. A., Foster, M. M., & Bennett, S. (2016). Challenges of user-centred assistive technology provision in Australia: Shopping without a prescription. *Disability and Rehabilitation: Assistive Technology*, *11*(3), 235-240. DOI: 10.3109/17483107.2014.941953
81. Sund, T., Iwarsson, S., Andersen, M. C., & Brandt, A. (2013). Documentation of and satisfaction with the service delivery process of electric powered scooters among adult users in different national contexts. *Disability and Rehabilitation: Assistive Technology*, *8*(2), 151-160. DOI: 10.3109/17483107.2012.699584
82. Ward, G., Fielden, S., Muir, H., Holliday, N., & Urwin, G. (2017). Developing the assistive technology consumer market for people aged 50-70. *Ageing and Society*, *37*(5), 1050-1067. DOI: 10.1017/S0144686X16000106
83. Clayback, D., Stanley, R., Leahy, J., Minkel, J., Piper, M., Smith, R. O., & Vaarwerk, T. (2014). *Standards for assistive technology funding: What are the right criteria?* Austin, TX: SEDL, Center on Knowledge Translation for Disability and Rehabilitation Research. Retrieved from http://ktdrr.org/ktlibrary/articles_pubs/Standards_for_Assistive_Technology_Funding.pdf
84. Flagg, J. L., Lockett, M. M., Condron, J., & Lane, J. P. (2015). Tools for analysis in assistive technology research, development and production. *Assistive Technology Outcomes and Benefits*, *9*(1), 20-37.
85. Lane, J. P. (2015). Aligning policy and practice in science, technology and innovation to deliver the intended socio-economic results: The case of assistive technology. *International Journal of Transitions and Innovative Systems*, *4*(3/4), 221-248.
86. Lane, J. P. (2015). Bridging the persistent gap between R&D and application: A historical review of government efforts in the field of assistive technology. *Assistive Technology Outcomes and Benefits*, *9*(1), 1-19.
87. Lane, J. P. (2017). *The case for industry leadership in science, technology and innovation (Sti) policy implementation for assistive technology.* RESNA Annual Conference, Arlington, VA.
88. Choi, Y. (2014). The costs and benefits of user input in design process: A practical guide for assistive technology device. *Archives of Design Research*, *27*(3), 17-35.
89. DOI: 10.15187/adr.2014.08.111.3.17

90. Bauer, S., & Buning, M. E. (Eds.). (2009). *The industry profile on wheeled mobility.* Buffalo, NY: RERC on Technology Transfer, University at Buffalo. Retrieved from https://www.ncart.us/uploads/userfiles/files/university-of-buffalo.pdf
91. Vanderheiden, G. C., Treviranus, J., Gemou, M., Bekiaris, E., Markus, K., Clark, C., & Basman, A. (2013). The Evolving Global Public Inclusive Infrastructure (GPII). In C. Stephanidis, & M. Antona (Eds.), *Universal access in human-computer interaction: Design methods, tools, and interaction techniques for eInclusion*. 7th International Conference, UAHCI 2013, Held as part of HCI International 2013, Las Vegas, NV, July 2013, Proceedings, Part I. Berlin, Germany: Springer.
92. Bauer, S. M., & Lane, J. P. (2006). Convergence of assistive devices and mainstream products: Keys to university participation in research, development and commercialization. *Technology and Disability*, *18*(2), 67-77.
93. Garçon, L., Khasnabis, C., Walker, L., Nakatani, Y., Lapitan, J., Borg, J., . . . Velazquez Berumen, A. (2016). Medical and assistive health technology: Meeting the needs of aging populations. *Gerontologist*, *56*(S2), S293-S302. DOI: 10.1093/geront/gnw005
94. Leahy, J. A. (2003). Paths to market for supply push technology transfer. *Journal of Technology Transfer*, *28*(3-4), 305-317. DOI: 10.1023/A:1024957231130
95. Lersilp, S., Putthinoi, S., & Okahashi, S. (2018). Information management for the assistive technology provision in community: Perspectives of local policymakers and health service providers. *Occupational Therapy International*, Article ID 8019283, 1-5. DOI: 10.1155/2018/8019283
96. Borg, J., Larsson, S., Östergren, P.-O., Rahman, A. S. M., Bari, N., & Khan, A. H. M. (2012). User involvement in service delivery predicts outcomes of assistive technology use: A cross-sectional study in Bangladesh. *BMC Health Services Research*, *12*(330), 1-10. DOI: 10.1186/1472-6963-12-330
97. Gibson, G., Newton, L., Pritchard, G., Finch, T., Brittain, K., & Robinson, L. (2016). The provision of assistive technology products and services for people with dementia in the United Kingdom. *Dementia*, *15*(4), 681-701. DOI: 10.1177/1471301214532643
98. Torrens, G. E. (2018). The order and priority of research and design method application within an assistive technology new product development process: A summative content analysis of 20 case studies. *Disability and Rehabilitation: Assistive Technology*, *13*(1), 66-77. DOI: 10.1080/17483107.2017.1280547
99. Wise, P. H. (2012). Emerging technologies and their impact on disability. *Future of Children*, *22*(1), 169-191.
100. Elsaesser, L.-J., & Bauer, S. (2012). Integrating medical, assistive, and universal design products and technologies: Assistive Technology Service Method (ATSM). *Disability and Rehabilitation: Assistive Technology*, *7*(4), 282-286. DOI: 10.3109/17483107.2011.635331
101. Kritikos, M. (2018). *Assistive technologies for people with disabilities; Part IV: Legal and socio-ethical perspectives.* Brussels, Belgium: Scientific Foresight Unit (STOA), European Parliament. Retrieved from http://www.europarl.europa.eu/RegData/etudes/IDAN/2018/603218/EPRS_IDA(2018)603218(ANN4)_EN.pdf
102. Tam, E., Mak, A. F. T., Chow, D., Wong, C., Kam, A., Luk, L., & Yuen, P. (2003). A survey on the need and funding for assistive technology devices and services in Hong Kong. *Journal of Disability Policy Studies*, *14*(3), 136-141.
103. Diaconu, K., Chen, Y.-F., Cummins, C., Moyao, G., Manaseki-Holland, S., & Lilford, R. (2017). Methods for medical device and equipment procurement and prioritization within low- and middle-income countries: Findings of a systematic literature review. *Globalization and Health*, *13*(59), 1-16. DOI: 10.1186/s12992-017-0280-2
104. Scherer, M. J., Craddock, G., & Mackeogh, T. (2011). The relationship of personal factors and subjective well-being to the use of assistive technology devices. *Disability and Rehabilitation*, *33*(10), 811-817. DOI: 10.3109/09638288.2010.511418
105. Carey, A. C., DelSordo, V., & Goldman, A. (2004). Assistive technology for all: Access to alternative financing for minority populations. *Journal of Disability Policy Studies*, *14*(4), 194-203.
106. Bonner, S., & Idris, T. (2012). *Assistive technology as a means of supporting people with dementia: A review.* London, England: Housing Learning and Improvement Network. Retrieved from https://www.housinglin.org.uk/Topics/type/Assistive-technology-as-a-means-of-supporting-people-with-dementia-A-Review/
107. Crossland, A., Ruedel, K., Gray, T., Wellington, D., Reynolds, J., & Perrot, M. (2016). *Future ready assistive technology: Fostering state supports for students with disabilities.* Washington, DC: Center on Technology and

Disability. Retrieved from https://www.ctdinstitute.org/library/2016-01-14/future-ready-assistive-technology-fostering-state-supports-students-disabilities

108. World Health Organization (GATE). (2014). *Global Cooperation on Assistive Health Technology; Meeting minutes.* Geneva, Switzerland: World Health Organization. Retrieved from http://www.who.int/phi/implementation/assistive_technology/gate_full_final_report_july_2014.pdf
109. Borg, J., Berman-Bieler, R., Khasnabis, C., Mitra, G., Myhill, W. N., & Raja, D. (2015). *Assistive technology for children with disabilities: Creating opportunities for education, inclusion and participation; A discussion paper.* World Health Organization & UNICEF. Retrieved from https://www.unicef.org/disabilities/files/Assistive-Tech-Web.pdf
110. Bardsley, G., Blocka, D., Borg, J., Brintnell, S., Constantine, D., Dillu, A., . . . Verhoeff, T. (2011). *Joint position paper on the provision of mobility devices in less-resourced settings: A step towards implementation of the Convention on the Rights of Persons with Disabilities (CRPD) related to personal mobility.* World Health Organization & USAID. Retrieved from http://www.who.int/disabilities/publications/technology/jpp_final.pdf
111. United Nations. (2006). *Convention on the Rights of Persons with Disabilities and Optional Protocol.* New York, NY: United Nations. Retrieved from https://www.un.org/development/desa/disabilities/convention-on-the-rights-of-persons-with-disabilities.html
112. MacLachlan, M. (2018). China, assistive technology and market shaping re Rise of a new superpower: Health and China's global trade ambitions. *BMJ*, *360*. DOI: 10.1136/bmj.k595
113. Eide, A. H., & *Øderud, T. (2009). Assistive technology in low-income countries. In M. MacLachlan, & L. Swartz (Eds.), Disability and international development: Towards inclusive global health. New York, NY: Springer.*
114. Visagie, S., Eide, A. H., Mannan, H., Schneider, M., Swartz, L., Mji, G., . . . MacLachlan, M. (2017). A description of assistive technology sources, services and outcomes of use in a number of African settings. *Disability and Rehabilitation: Assistive Technology*, *12*(7), 705-712. DOI: 10.1080/17483107.2016.1244293
115. Owuor, J., Larkan, F., Kayabu, B., Fitzgerald, G., Sheaf, G., Dinsmore, J., . . . MacLachlan, M. (2018). Does assistive technology contribute to social inclusion for people with intellectual disability? A systematic review protocol. *BMJ Open*, *8*(2), e017533. DOI: 10.1136/bmjopen-2017-017533
116. Boot, F. H., Dinsmore, J., Khasnabis, C., & MacLachlan, M. (2017). Intellectual disability and assistive technology: Opening the GATE wider. *Frontiers in Public Health*, *5*(10), 1-4. DOI: 10.3389/fpubh.2017.00010

© 2018 by the authors. Submitted for possible open access publication under the terms and conditions of the Creative Commons Attribution (CC BY) license (http://creativecommons.org/licenses/by/4.0/).
